# Supplementary material for: Lower alpha, higher beta, and similar gamma diversity of saproxylic beetles in unmanaged compared to managed Norway spruce stands
Source: PLoS One. 2022 Jul 8;17(7):e0271092. doi: 10.1371/journal.pone.0271092 (PMC9269974; doi:10.1371/journal.pone.0271092)
Supplement: S1 Fig — (PDF) [file pone.0271092.s001.pdf]

S1 Figs

Rank abundance curves

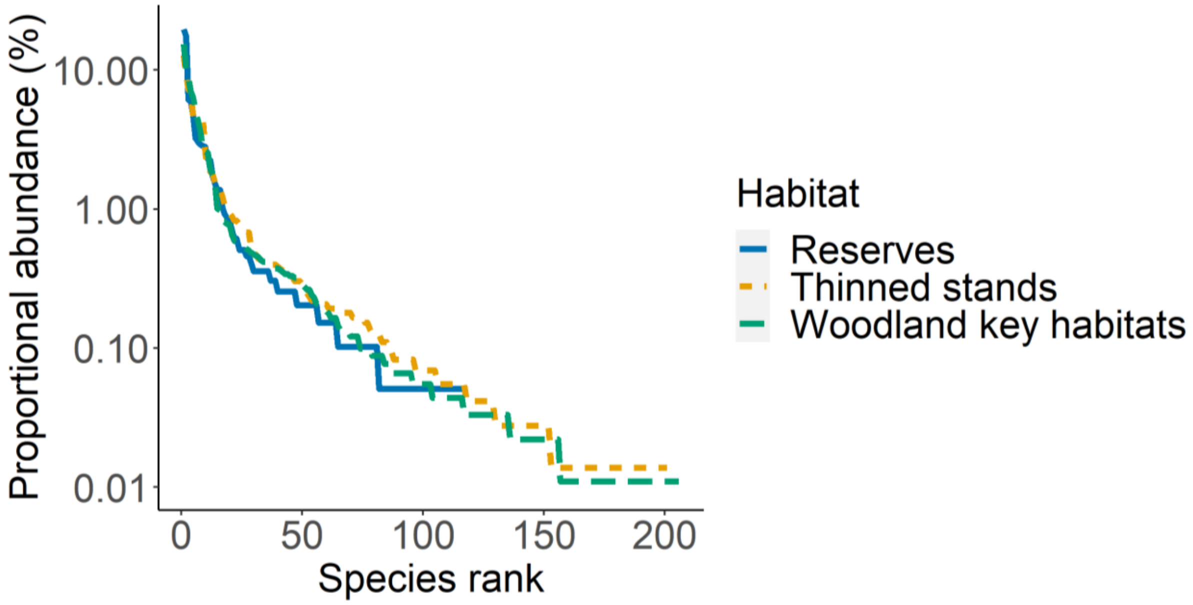

S1 Fig 1. Rank abundance curves for habitats based on the 2017 Jönköping sample.

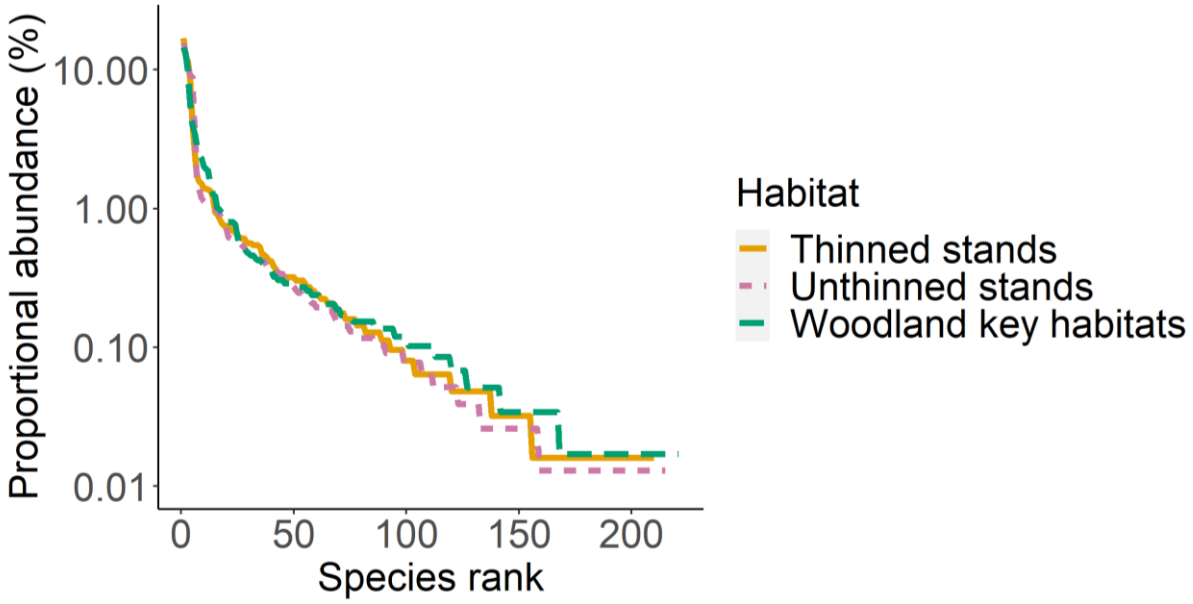

S1 Fig 2. Rank abundance curves for habitats based on the 2018 Örebro sample.
