# Supplementary material for: Lower alpha, higher beta, and similar gamma diversity of saproxylic beetles in unmanaged compared to managed Norway spruce stands
Source: PLoS One. 2022 Jul 8;17(7):e0271092. doi: 10.1371/journal.pone.0271092 (PMC9269974; doi:10.1371/journal.pone.0271092)
Supplement: S1 Table — (PDF) [file pone.0271092.s002.pdf]

S2 Table

PERMDISP based on Sørensen dissimilarity

S2 Table. Multivariate dispersion (mean distances from centroid) for traps of each forest type, from PERMDISP analysis based on Sørensen dissimilarity of saproxylic species presence/absence data. P-values below 0.05 in bold.

			Pairwise comparisons		
	**Mean**	**SE**	**Reserve**	**Thinned**	**Unthinned**
**WKH**	37.7	0.75	t = 4.4; p = **0.001**	t = 3.1; p = **0.002**	t = 2.9; p = **0.012**
**Reserve**	28.9	0.89		t = 4.0; p = **0.006**	t = 2.2; p = 0.125
**Thinned**	34.8	0.55			t = 0.9; p = 0.432
**Unthinned**	33.8	1.17			
